# Live-streaming interaction and young consumers’ identification with time-honored brands: The roles of emotional arousal and relationship strength

**DOI:** 10.1371/journal.pone.0358753

**Published:** 2026-09-24

**Authors:** Tingting Zhu, Yuhuan Wang

**Affiliations:** School of Business, Anhui University of Technology, Ma’anshan, Anhui, People’s Republic of China; Sichuan Agricultural University, CHINA

## Abstract

Time-honored brands, as carriers of cultural heritage and traditional craftsmanship, face significant challenges in achieving sustainable development in the digital era. With the rapid growth of e-commerce live streaming and the increasing dominance of younger consumers, how these brands can effectively engage youth segments and enhance brand identification has become a critical issue. Drawing on the stimulus–organism–response (SOR) framework and brand authenticity theory, this study examines the mechanisms through which product interaction and interpersonal interaction influence young consumers’ identification with time-honored brands in live-streaming contexts. Data were collected from 415 consumers aged 18–40 who had watched live-streaming sessions of time-honored brands, and the proposed model was tested using partial least squares structural equation modeling (PLS-SEM). The results showed that product interaction and interpersonal interaction were positively associated with social presence and perceived brand authenticity. Both social presence and perceived brand authenticity were positively associated with emotional arousal, which was in turn associated with brand identification. Both interaction types also showed significant indirect associations with brand identification through two serial pathways: (1) social presence → emotional arousal and (2) perceived brand authenticity → emotional arousal. The interactions of consumer–streamer relationship strength with social presence and perceived brand authenticity were not statistically significant. This study contributes to the literature by developing an integrated framework that combines SOR theory and brand authenticity theory, and by uncovering the dual emotional mechanisms underlying brand identification in e-commerce live-streaming environments. The findings provide new insights into how interactive digital platforms can facilitate the revitalization of time-honored brands and strengthen their connection with younger consumers.

## 1. Introduction

Time-honored brands refer to firms or brand entities that possess a long history, stable product quality, and enduring cultural significance, often recognized for their transmission of traditional craftsmanship, heritage, and commercial reputation across generations [1]. As such, they represent an important component of cultural heritage, embodying accumulated historical value, artisanal expertise, and symbolic meaning. Beyond their economic function, time-honored brands serve as carriers of cultural identity and repositories of collective memory, thereby contributing to the preservation of cultural continuity and the enhancement of cultural soft power.

Despite their cultural and symbolic importance, many time-honored brands face substantial challenges in the contemporary marketplace. Prior research indicates that a significant proportion of such firms have experienced long-term financial difficulties and declining competitiveness, highlighting a pronounced imbalance between their intrinsic cultural value and their current market performance [2]. This tension between heritage preservation and market adaptation has attracted growing attention from both academia and practice.

Existing studies have primarily focused on the revitalization of time-honored brands, suggesting that strategies such as communication innovation [3–5], cross-industry collaboration [6,7], authenticity construction [8–10], and archival and cultural resource development [11,12] can enhance consumer cognition, identification, and loyalty. While these studies provide valuable insights, they are largely situated within traditional or static marketing contexts.

With the rapid development of digital technologies such as mobile internet and artificial intelligence, new marketing formats have emerged, among which e-commerce live streaming has become one of the most influential. By integrating real-time video, interactive communication, and online transactions, live streaming offers consumers a novel and immersive shopping experience. Its rapid growth has made it a key channel in the digital commerce ecosystem. Nevertheless, how time-honored brands can effectively leverage this emerging format remains an open question [13,14]. For time-honored brands, however, live streaming is a potentially double-edged channel. Its fast-paced, entertainment-oriented, promotion-intensive, and often discount-driven format can increase visibility and engagement, but it may also conflict with the historical continuity, craftsmanship, and cultural integrity on which these brands’ authenticity depends. When live-streaming presentations rely excessively on price promotions, scripted excitement, or urgent calls to purchase, consumers may perceive heritage as being instrumentalized for short-term sales, thereby weakening rather than reinforcing perceived brand authenticity. Conversely, transparent product demonstrations, credible heritage storytelling, and responsive interaction may make traditional brand meanings more accessible to younger consumers. The central issue is therefore not simply whether time-honored brands should adopt live streaming, but how they can use it without diluting the authenticity that differentiates them.

Against this background, existing research on live streaming has primarily examined immediate consumer responses, such as purchase intention and decision-making processes [15–17], while paying relatively limited attention to whether commercially intensive live-streaming practices preserve or undermine heritage-based brand meanings. In particular, two connected mechanisms remain insufficiently understood. First, it is unclear how consumers use live-streaming interactions to evaluate whether a time-honored brand’s contemporary presentation remains genuine and consistent with its historical identity. Second, the way in which this authenticity evaluation, together with the social immediacy of live streaming, generates emotional arousal and ultimately shapes brand identification has not been sufficiently explored, especially among younger consumers [18]. Brand identification is particularly relevant here because it represents a longer-term, affect-laden consumer–brand relationship rather than an immediate transactional response.

In this study, young consumers were operationally defined as adults aged 18–40, who constitute the primary user base of e-commerce live-streaming platforms and are typically characterized by high digital literacy, strong engagement with interactive media, and a preference for experiential and emotionally driven consumption [19]. This group is particularly relevant in the context of live-streaming commerce, as they are more responsive to real-time interaction, social cues, and immersive shopping experiences. Despite their growing importance, there remains a lack of systematic understanding of how live-streaming interactions can effectively trigger emotional resonance and foster identification with time-honored brands among this segment.

To address these gaps, this study addresses three research questions: (1) How are product interaction and interpersonal interaction associated with young consumers’ identification with time-honored brands in e-commerce live-streaming contexts? (2) What serial mediating roles do the social presence–emotional arousal and perceived brand authenticity–emotional arousal pathways play in these associations? (3) Does consumer–streamer relationship strength moderate the associations of social presence and perceived brand authenticity with emotional arousal?

Guided by these questions, the study develops an integrated framework based on the SOR model and brand authenticity theory. Within this framework, perceived brand authenticity is treated not merely as an additional explanatory variable, but as a meaning-based evaluation of whether a commercially oriented live-streaming presentation remains consistent with a brand’s historical identity, cultural values, and craftsmanship. The framework distinguishes an experiential pathway involving social presence and emotional arousal from an authenticity-based pathway involving perceived brand authenticity and emotional arousal.

Empirically, this study is based on 415 valid responses collected from consumers aged 18–40 who had engaged with live-streaming sessions of time-honored brands within the past six months. By adopting a PLS-SEM approach, the study evaluates the proposed experiential and authenticity-based pathways through which live-streaming interactions are associated with brand identification. In doing so, it moves beyond the assumption that digital engagement is uniformly beneficial and clarifies why the preservation of perceived authenticity is theoretically central to the use of live streaming by time-honored brands. This perspective contributes to a more nuanced understanding of how interactive digital environments can support the sustainable development of time-honored brands in the digital era.

## 2. Theoretical framework and research hypotheses

### 2.1. Theoretical framework

#### 2.1.1. SOR theory (stimulus–organism–response framework).

The SOR framework provides a foundational lens for understanding how environmental cues shape individual cognition and behavior. Originating from environmental psychology and formalized by Mehrabian and Russell (1974) [20], the framework conceptualizes consumer behavior as a process in which external stimuli trigger internal psychological states, which subsequently lead to behavioral outcomes. In digital commerce environments, particularly in e-commerce live streaming, this framework has been widely employed to explain how interactive and immersive stimuli influence consumer engagement and decision-making processes [21,22].

Existing applications of the SOR framework in live-streaming contexts largely follow a standardized logic: platform-generated stimuli enhance consumers’ affective and cognitive states, which in turn drive behavioral responses. While this approach effectively captures the dynamics of real-time interaction and emotional engagement, it predominantly assumes that consumer responses are shaped by immediate situational cues. As such, it places primary emphasis on short-term experiential factors and often overlooks the role of historically embedded brand meanings.

This limitation becomes particularly salient in the context of time-honored brands. Unlike emerging or purely commercial brands, time-honored brands are deeply rooted in cultural heritage, historical continuity, and symbolic value. Their market performance is not solely determined by real-time stimuli but is also shaped by consumers’ interpretations of enduring brand meanings [23]. Consequently, a purely stimulus-driven explanation is insufficient to capture the complexity of consumer responses in this context. What is required is a theoretical extension that accounts for how historically accumulated brand value interacts with situational stimuli in shaping consumer perception and behavior.

#### 2.1.2. Brand authenticity theory.

Brand authenticity theory provides a critical complementary perspective by addressing the interpretive processes through which consumers evaluate brand meaning. Rather than focusing solely on immediate reactions to external stimuli, this perspective emphasizes the extent to which a brand is perceived as genuine, consistent, and faithful to its historical and cultural essence [1,23]. Authenticity, in this sense, is not an inherent attribute but a consumer-based evaluation shaped by both past associations and present experiences.

For time-honored brands, authenticity constitutes a central dimension of brand value. These brands derive their legitimacy not only from product functionality but also from their ability to embody tradition, craftsmanship, and cultural continuity [23]. Consumers’ engagement with such brands therefore involves a process of meaning verification—assessing whether contemporary brand expressions remain aligned with historically established identities. This evaluative process cannot be fully explained within the traditional SOR framework, as it extends beyond immediate psychological reactions to encompass deeper symbolic interpretation.

Within e-commerce live streaming, this tension can be conceptualized as a commercialization–heritage paradox. Live-streaming commerce is organized around immediacy, high-energy presentation, flash discounts, scarcity cues, and rapid conversion [24,25]. Time-honored brands, by contrast, derive authenticity from historical continuity, stable quality, traditional craftsmanship, and cultural narratives accumulated over time [1,23]. The conflict arises because hard-selling practices can compress complex heritage meanings into short-term promotional claims. When historical symbols or craftsmanship narratives are used mainly to create urgency or justify discounts, consumers may interpret the presentation as staged or opportunistic rather than as a credible expression of the brand’s enduring identity. Perceived inconsistency between contemporary commercial conduct and inherited brand values may therefore weaken authenticity evaluations.

This paradox does not mean that commercialization inevitably undermines authenticity. Live streaming can also make heritage visible and verifiable through transparent product demonstrations, credible explanations of production processes, and responsive storytelling [23,25]. Consumers thus assess whether heritage cues provide substantive evidence of continuity, sincerity, and craftsmanship or merely function as decorative sales devices. This authenticity dilemma requires consumers to reconcile a fast-paced commercial encounter with expectations of historical and cultural consistency, explaining why perceived brand authenticity constitutes a distinct meaning-based mechanism in the present framework.

#### 2.1.3. Integrated theoretical framework.

The integration of the SOR framework with brand authenticity theory enables a more comprehensive explanation of consumer behavior in this context. Specifically, it extends the SOR logic by incorporating a meaning-based evaluative layer between environmental stimuli and consumer responses. This integration shifts the focus from a purely reactive process to a dual-process mechanism in which consumers both experience and interpret stimuli.

From this perspective, live-streaming environments do not simply generate affective or cognitive reactions; they also create vivid and socially meaningful mediated experiences [26–30] and serve as arenas in which brand meaning is dynamically constructed and assessed. The effectiveness of such environments depends not only on their ability to engage consumers but also on their capacity to convey and reinforce authentic brand narratives [1]. For time-honored brands, this implies that consumer responses are contingent upon whether live-streaming practices are perceived as consistent with the brand’s historical identity and cultural values.

Within this integrated framework, product interaction and interpersonal interaction are conceptually distinct stimulus categories. Product interaction is object-centered and task-oriented: it concerns the focal product and provides product-related evidence through demonstrations, close-up displays of materials or craftsmanship, explanations of use and technology, and responses to product-focused questions [31]. Its proximal function is to increase informational diagnosticity and support verification of whether a time-honored brand’s current offerings remain consistent with its claimed quality, craftsmanship, and heritage.

Interpersonal interaction is person-centered and relationship-oriented: it comprises reciprocal exchanges between the streamer and viewers and among viewers, including live chat, personalized responses, reactions to streamer prompts, and shared discussion [25,29]. Its proximal function is to make other participants salient, responsive, and psychologically accessible, thereby strengthening social presence. The boundary is functional rather than tied to a specific platform feature: live chat constitutes product interaction when used to clarify product attributes, but interpersonal interaction when used to create reciprocal recognition, social connection, or awareness of co-viewers.

The two stimuli are therefore complementary but not interchangeable. Product interaction is expected to operate most directly through authenticity verification, whereas interpersonal interaction is expected to operate most directly through social presence. Cross-path effects remain theoretically plausible because product-focused exchanges are delivered by responsive human actors, and interpersonal exchanges can shape the credibility and sincerity of heritage communication. This distinction provides the basis for the hypotheses developed below.

**Fig 1:**
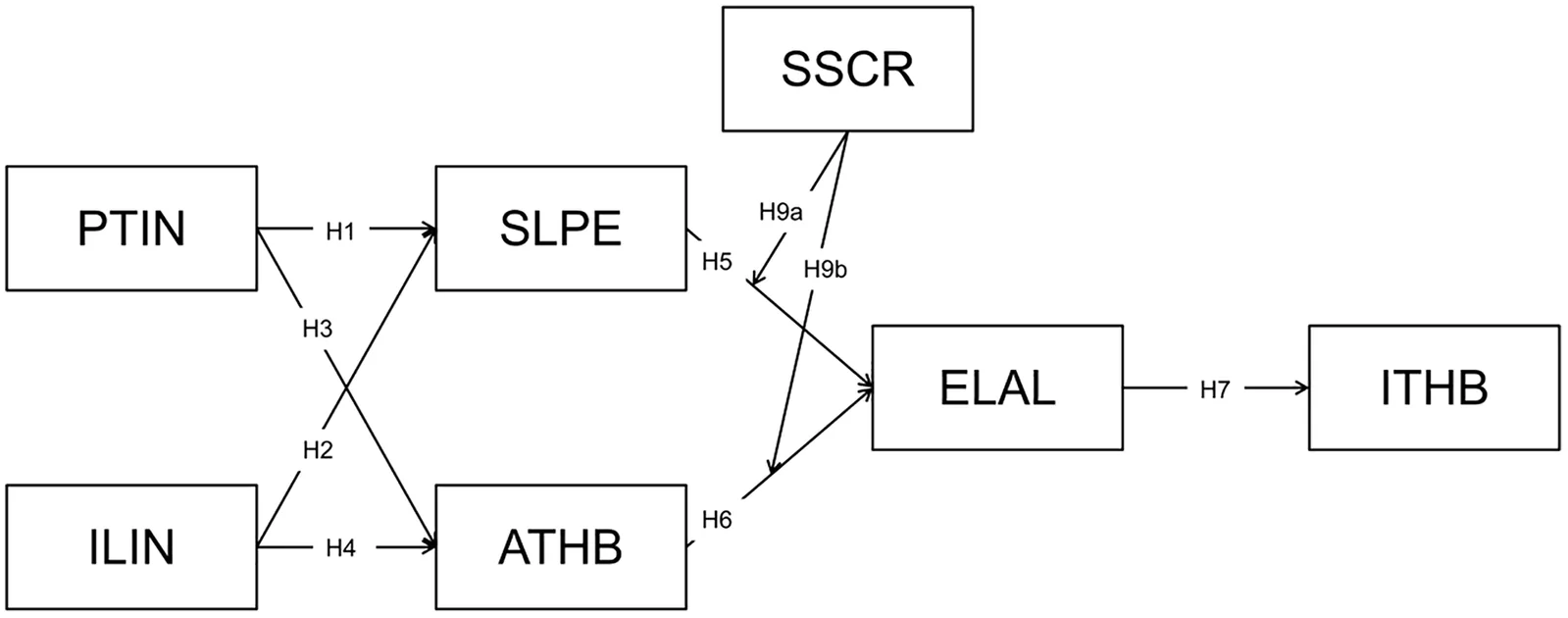
Conceptual model. PTIN = Product Interaction; ILIN = Interpersonal Interaction; SLPE = Social presence; ATHB = Perceived authenticity of time-honored brands; ELAL = Emotional Arousal; SSCR = Consumer–streamer relationship strength; ITHB = Brand identification with time-honored brands.

Taken together, the integrated framework specifies product interaction and interpersonal interaction as the external stimuli (S); social presence and perceived brand authenticity as parallel first-stage organismic states, followed by emotional arousal as a second-stage organismic state (O); and identification with time-honored brands as the response (R). Consumer–streamer relationship strength is modeled as a boundary condition on the relationships between social presence and emotional arousal and between perceived brand authenticity and emotional arousal. As illustrated in Fig 1, the framework therefore comprises two complementary serial pathways: product/interpersonal interaction → social presence → emotional arousal → brand identification, and product/interpersonal interaction → perceived brand authenticity → emotional arousal → brand identification. Fig 1 provides an early visual guide to the hypotheses developed in Section 2.2.

### 2.2. Research hypotheses

E-commerce live streaming combines object-centered product presentation with person-centered social exchange [24,25]. Following the distinctions above, this study treats product interaction and interpersonal interaction as separate external stimuli within the SOR framework rather than as interchangeable indicators of general interactivity.

The hypothesized paths reflect both their primary and secondary functions. Product interaction primarily supports the verification of product and heritage claims, whereas interpersonal interaction primarily supports social presence through reciprocity and co-presence. At the same time, product-focused exchange may enhance social presence through real-time human responsiveness, and interpersonal exchange may influence authenticity through credible storytelling and social validation. The following subsections develop these pathways separately.

#### 2.2.1. The impact of live-streaming interaction on social presence.

*The impact of product interaction on social presence:* Product interaction is an object-centered, task-oriented stimulus involving product-focused presentation and information exchange. It includes demonstrations of product use and technology, close-up or multi-angle displays of materials and craftsmanship, explanations of product features, and real-time responses to product-related questions [31]. Its defining function is to increase the diagnosticity of product information; it is therefore distinct from interpersonal interaction, which is organized around social exchange and relationship building.

Social presence refers to the extent to which other participants are experienced as real, accessible, and responsive within a mediated environment [28–30]. Although product interaction is not primarily relational, it can contribute to social presence when product information is delivered contingently by a visible and responsive streamer.

Dynamic demonstrations and immediate answers signal that a human communicator is present and reacting in real time. This responsiveness reduces the distance associated with mediated shopping and makes the live-streaming setting feel more immediate and inhabited [32,33]. Thus, the product interaction-social presence link arises from the human responsiveness embedded in product-focused exchange, rather than from social bonding itself.

For time-honored brands, live demonstrations of materials, production techniques, or craftsmanship can make the encounter especially vivid and allow viewers to feel that they are observing both the product and its presenter in a shared setting. Product interaction may therefore provide a secondary experiential route to social presence, even though its more direct theoretical role is authenticity verification.

Accordingly, product interaction is expected to strengthen perceived social presence through the immediacy and contingent responsiveness of product-focused communication. The following hypothesis is proposed:

**H1**. *Product interaction has a positive effect on consumers’ perceived social presence.*

*The impact of interpersonal interaction on social presence.* Interpersonal interaction is a person-centered, relationship-oriented stimulus comprising reciprocal exchanges between the streamer and viewers and among viewers. It includes live chat, personalized responses, greetings, question-and-answer exchanges, reactions to streamer prompts, and shared discussion [25,29,34,35]. Unlike product interaction, its defining function is not the verification of product attributes but the creation of reciprocal recognition and social connection.

Interpersonal interaction is therefore directly aligned with social presence. Immediate feedback, personal acknowledgment, and visible exchanges among co-viewers make other participants salient, accessible, and psychologically real [30,32].

Synchronous comments, bullet-screen messages, and collective reactions also allow viewers to recognize that others are watching and responding at the same time. This awareness of co-viewers supports co-presence, sociability, and a sense of community [33,36].

For time-honored brands, streamers may communicate heritage stories during these exchanges, but the effect on social presence derives from how the communication occurs: viewers can respond, receive recognition, and observe others’ reactions. Interpersonal interaction thus represents the more proximal social-presence stimulus.

Accordingly, interpersonal interaction is expected to strengthen social presence by increasing relational immediacy, reciprocity, and awareness of co-viewers. The following hypothesis is proposed:

**H2**. *Interpersonal interaction has a positive effect on consumers’ perceived social presence.*

#### 2.2.2. The impact of live-streaming interaction on the perceived authenticity of time-honored brands.

*The impact of product interaction on the perceived authenticity of time-honored brands.* Perceived brand authenticity reflects whether a brand is regarded as genuine, consistent, and faithful to its historical origins and core values [23,37]. For time-honored brands, this judgment depends partly on whether contemporary products provide credible evidence of historical continuity, stable quality, and traditional craftsmanship.

Product interaction is theoretically central to this evaluation because it supplies product-based evidence that consumers can inspect. Detailed demonstrations, close-up displays of materials and craftsmanship, explanations of product use or production techniques, and responses to product-focused questions increase informational transparency and diagnosticity [25,31].

These cues allow consumers to compare heritage claims with observable product attributes. Demonstrating a traditional process or explaining why a material or technique has been retained can make heritage verifiable rather than merely rhetorical. Product interaction therefore directly supports meaning verification by showing whether the brand’s current offering is consistent with its inherited identity [1,23].

This evidence-based function is especially important under the commercialization–heritage paradox. When live streams are highly promotional, detailed and transparent product presentation can counter the impression that heritage is being used only as a sales device.

By reducing information asymmetry and enabling direct verification, product interaction provides a proximal diagnostic route to perceived authenticity [38].

Accordingly, product interaction is expected to strengthen perceived authenticity by making quality, craftsmanship, and heritage claims more observable and assessable. The following hypothesis is proposed:

**H3.** *Product interaction has a positive effect on consumers’ perceived authenticity of time-honored brands.*

*The impact of interpersonal interaction on the perceived authenticity of time-honored brands.* Interpersonal interaction may also shape authenticity, but through a mechanism distinct from product-based verification. As a person-centered stimulus, it provides relational and source-based cues regarding the sincerity, credibility, and consistency of brand communication [1,25].

Through real-time dialogue, personalized responses, and heritage storytelling, streamers can explain the brand’s values and historical narratives in a form that viewers can question and evaluate [23]. Responsive communication may appear less scripted than one-way promotion and can therefore strengthen perceptions that the brand is communicating honestly.

Interactions among viewers provide an additional source of social validation. Comments and shared discussion expose brand claims to collective scrutiny, allowing consumers to observe whether other viewers corroborate, question, or elaborate on the brand narrative [35,36]. These exchanges can influence whether heritage communication is perceived as sincere and consistent rather than opportunistic.

Interpersonal interaction thus supports authenticity indirectly through source credibility, meaning co-construction, and social validation; it does not primarily verify product attributes.

This relational route complements the product-evidence route of product interaction and is particularly relevant when authenticity depends on the credible communication of historical and cultural meanings [38].

Accordingly, interpersonal interaction is expected to strengthen perceived authenticity by increasing the credibility and sincerity of heritage communication. The following hypothesis is proposed:

**H4.** *Interpersonal interaction has a positive effect on consumers’ perceived authenticity of time-honored brands.*

#### 2.2.3. The impacts of social presence and brand authenticity on emotional arousal.

*The impact of social presence on emotional arousal.* Within the SOR framework, consumers’ internal psychological states serve as critical mediating mechanisms through which external stimuli influence behavioral outcomes. Social presence, as a key organismic state, reflects the extent to which consumers perceive others as psychologically real and emotionally accessible within mediated environments [28,30]. In e-commerce live-streaming contexts, social presence represents an important experiential outcome of interactive stimuli, capturing how consumers internalize the social and immersive characteristics of the environment.

Prior research suggests that environments characterized by high levels of social presence enhance emotional engagement by creating a sense of immediacy, intimacy, and co-experience [32,33]. When consumers perceive that they are interacting with real and responsive others, the mediated environment becomes more vivid and socially rich, which intensifies their emotional reactions. This aligns with the SOR logic, whereby enriched environmental cues are translated into affective responses through internal psychological processes.

In the context of e-commerce live streaming, social presence is fostered through real-time interaction, visual immersion, and synchronous communication. These features collectively create a dynamic environment in which consumers feel actively involved rather than passively observing. Such involvement enhances emotional stimulation by increasing perceived participation and reducing psychological distance between consumers and the shopping context [25,36]. As a result, consumers are more likely to experience heightened levels of emotional arousal, including excitement, pleasure, and enthusiasm.

Moreover, social presence facilitates emotional contagion and shared experiences among participants. When consumers observe others’ reactions or engage in collective interaction, they are more likely to align their emotional states with those of the group, thereby amplifying their own emotional responses [33]. This mechanism is particularly salient in live-streaming environments, where synchronous communication enables immediate feedback and collective emotional expression.

In the context of time-honored brands, this process acquires additional significance. As these brands are associated with cultural heritage and symbolic meaning, consumers’ emotional responses are not only driven by situational stimuli but also by their interpretation of brand-related cues. When a strong sense of social presence is established, consumers become more receptive to such cues, allowing them to experience deeper emotional resonance with the brand. This enhances the intensity of emotional arousal and strengthens affective engagement.

Therefore, integrating the SOR framework with the authenticity perspective suggests that social presence functions as a key internal mechanism through which interactive environments translate into emotional responses. By enhancing immersion, relational connection, and shared experience, social presence significantly increases consumers’ emotional arousal in e-commerce live-streaming contexts. Accordingly, the following hypothesis is proposed:

**H5.** *Social presence in e-commerce live streaming has a positive effect on consumers’ emotional arousal.*

*The impact of brand authenticity on emotional arousal.* Within the extended SOR framework, consumers’ emotional responses are not only shaped by environmental stimuli and experiential states but are also influenced by their interpretation of brand meaning. Brand authenticity, as a central evaluative construct, reflects the extent to which a brand is perceived as genuine, consistent, and faithful to its historical origins and core values [1,23]. In this sense, authenticity represents a meaning-based cognitive appraisal that can trigger affective reactions.

Prior research suggests that authenticity is closely associated with consumers’ emotional responses, as it fosters perceptions of sincerity, credibility, and trustworthiness [38]. When consumers perceive a brand as authentic, they are more likely to experience positive emotions such as pleasure, attachment, and psychological comfort. This relationship is particularly relevant in experiential consumption contexts, where emotional engagement plays a central role in shaping consumer responses [37].

In e-commerce live-streaming environments, brand authenticity is dynamically constructed through interactive presentations and real-time communication. Unlike static online contexts, live streaming enables consumers to observe and evaluate brand-related cues—such as production processes, craftsmanship, and storytelling—in a vivid and immediate manner. These cues allow consumers to assess whether the brand’s current representation aligns with its perceived heritage and identity. When such alignment is achieved, consumers experience a sense of coherence and credibility, which enhances their emotional engagement with the brand [1].

For time-honored brands, this mechanism is particularly salient. These brands are inherently associated with historical continuity, cultural heritage, and symbolic meaning. As a result, consumers’ perceptions of authenticity often evoke deeper emotional responses, including nostalgia, cultural resonance, and identity-related feelings [23]. When authenticity is effectively communicated in live-streaming contexts, it not only reinforces cognitive evaluations but also stimulates affective reactions by connecting consumers with the brand’s historical and cultural narratives.

Moreover, authenticity can amplify emotional arousal by enhancing perceived value and reducing uncertainty. When consumers perceive that a brand remains faithful to its origins and maintains consistent quality, they are more likely to feel confident and emotionally invested in the brand [38]. This process transforms authenticity from a purely cognitive judgment into an affective driver that intensifies emotional arousal.

Therefore, integrating the SOR framework with brand authenticity theory suggests that authenticity functions as a critical meaning-based mechanism that translates brand-related evaluations into emotional responses. In the context of time-honored brands, authenticity not only reinforces trust and credibility but also evokes culturally embedded emotional experiences, thereby significantly enhancing consumers’ emotional arousal. Accordingly, the following hypothesis is proposed:

**H6.** *The perceived authenticity of time-honored brands in e-commerce live streaming has a positive effect on consumers’ emotional arousal.*

#### 2.2.4. The impact of emotional arousal on brand identification with time-honored brands.

Within the SOR framework, emotional arousal represents a core organismic state through which environmental stimuli are translated into behavioral and attitudinal responses. Emotional arousal refers to the intensity of affective reactions experienced by individuals in response to external stimuli, encompassing feelings such as excitement, pleasure, and enthusiasm [20,39]. In experiential consumption contexts, such emotional responses play a critical role in shaping consumers’ evaluations and subsequent behavioral intentions.

Brand identification reflects the extent to which consumers perceive a sense of connection, attachment, and self-relevance with a brand [40]. It represents a deeper level of consumer–brand relationship that goes beyond functional evaluation, involving emotional bonding and psychological alignment. Prior research suggests that emotional experiences serve as key antecedents of brand identification, as positive affect enhances consumers’ attachment and strengthens their sense of belonging to the brand [41].

In e-commerce live-streaming environments, emotional arousal is intensified by real-time interaction, vivid presentation, and immersive communication. These features create an engaging and dynamic context in which consumers experience heightened emotional responses. According to the SOR logic, such emotional arousal acts as a mediating mechanism that links interactive stimuli and experiential perceptions to subsequent relational outcomes. When consumers experience strong emotional reactions during live streaming, they are more likely to form favorable attitudes and develop a deeper psychological connection with the brand [25,36].

For time-honored brands, this mechanism is particularly pronounced. These brands are embedded with cultural heritage, historical continuity, and symbolic meaning, which can evoke richer and more enduring emotional responses. When consumers experience emotional arousal in response to brand-related cues—such as storytelling, craftsmanship demonstrations, and cultural narratives—they are more likely to internalize these meanings and integrate them into their self-concept [23]. This process strengthens consumers’ sense of identity alignment with the brand, thereby enhancing brand identification.

Moreover, emotional arousal reinforces brand identification by increasing engagement and reducing psychological distance. When consumers feel emotionally stimulated, they become more involved in the consumption experience and more receptive to brand-related messages. This heightened engagement fosters a sense of intimacy and belonging, which are critical components of brand identification [40]. In live-streaming contexts, where interaction is immediate and participatory, such effects are further amplified.

Therefore, integrating the SOR framework with the authenticity perspective suggests that emotional arousal functions as a key affective mechanism that transforms experiential and meaning-based evaluations into relational outcomes. In the context of time-honored brands, emotional arousal not only enhances consumers’ engagement but also deepens their psychological connection with the brand, ultimately strengthening brand identification. Accordingly, the following hypothesis is proposed:

**H7**. *Emotional arousal in e-commerce live streaming has a positive effect on consumers’ identification with time-honored brands.*

#### 2.2.5. Serial indirect effects.

*Serial indirect effect through social presence and emotional arousal for product interaction*. Within the SOR framework, consumer responses are not generated through a single-stage process but rather emerge through a sequence of interrelated psychological states. In e-commerce live-streaming environments, interactive features function as external stimuli that first shape consumers’ experiential perceptions and subsequently trigger affective responses, ultimately influencing relational outcomes. This suggests the existence of a serial indirect effect linking environmental stimuli to consumer responses.

Product interaction, as a key stimulus in live-streaming contexts, enhances the vividness, interactivity, and informational richness of the environment. Through real-time demonstrations, dynamic presentations, and immediate feedback, consumers are able to experience a more immersive and engaging shopping process [25,31]. Such experiential enhancement reduces psychological distance and strengthens consumers’ perception of being present within the mediated environment. As a result, product interaction contributes to the formation of social presence, which represents the first-stage organismic response in the SOR process [30,32].

Social presence, in turn, plays a critical role in shaping consumers’ emotional responses. When consumers perceive a high level of co-presence and interaction with others, the shopping environment becomes more vivid and socially meaningful, thereby intensifying emotional engagement [33,36]. This process reflects a transition from cognitive immersion to affective activation, in which the perceived realism and immediacy of the environment stimulate emotional arousal.

Emotional arousal further serves as a key driver of relational outcomes, particularly brand identification. As an affective state, emotional arousal enhances consumers’ engagement and facilitates the internalization of brand-related meanings, thereby strengthening their psychological connection with the brand [39,40]. In this sense, emotional arousal represents a second-stage organismic response that bridges experiential perception and attitudinal outcomes.

For time-honored brands, this sequential mechanism is particularly salient. These brands are characterized by rich historical and cultural connotations, which require not only cognitive understanding but also affective engagement for effective transmission. Product interaction in live-streaming environments provides a means to present such heritage-related cues in a vivid and accessible manner. Through the enhancement of social presence, consumers are able to experience these cues in a more immersive context, which subsequently evokes emotional arousal and facilitates deeper brand identification.

Therefore, integrating the SOR framework with the experiential and affective perspectives suggests a sequential mediation process in which product interaction influences brand identification through the successive effects of social presence and emotional arousal. This serial indirect effect highlights how interactive stimuli are translated into relational outcomes via multiple stages of psychological processing. Accordingly, the following hypothesis is proposed:

**H8a.** *Product interaction positively affects consumers’ identification with time-honored brands through the sequential mediating effects of social presence and emotional arousal.*

*Serial indirect effect through social presence and emotional arousal for interpersonal interaction.* Within the SOR framework, interpersonal interaction in e-commerce live-streaming environments represents a socially embedded stimulus that shapes consumers’ internal psychological states through a multi-stage process. Unlike product interaction, which primarily enhances informational richness, interpersonal interaction emphasizes relational communication, emotional exchange, and social connection, thereby exerting a stronger influence on consumers’ experiential and affective responses [25,29].

Interpersonal interaction contributes to the formation of social presence by enhancing the perceived immediacy, intimacy, and responsiveness of the communication environment. Through real-time dialogue, personalized feedback, and synchronous communication, consumers perceive others as psychologically real and socially accessible [30,32]. This process reduces perceived distance between participants and transforms the live-streaming context into a socially immersive environment, thereby strengthening the perception of co-presence. As the first-stage organismic response, social presence captures how consumers internalize relational cues embedded in interactive environments.

Building upon social presence, emotional arousal emerges as a second-stage organismic response. When consumers experience a strong sense of co-presence, they are more likely to engage emotionally with the live-streaming content and with other participants. Prior research indicates that socially rich environments facilitate emotional contagion and shared affective experiences, which intensify consumers’ emotional responses [33,36]. In this process, the transition from perceived social connection to affective activation reflects a shift from cognitive immersion to emotional engagement.

For time-honored brands, this sequential mechanism is particularly important. These brands rely heavily on cultural narratives, historical continuity, and symbolic meaning to establish consumer connections. Interpersonal interaction enables streamers to convey these elements through storytelling and emotional communication, allowing consumers to interpret and experience the brand’s cultural value in a socially embedded context [23]. When such meaning-based cues are processed within an environment characterized by strong social presence, they are more likely to evoke emotional resonance, including feelings of nostalgia, attachment, and cultural identification.

Emotional arousal, in turn, facilitates the formation of brand identification by enhancing consumers’ engagement and strengthening their psychological connection with the brand [39,40]. As an affective mechanism, it enables consumers to internalize brand-related meanings and integrate them into their self-concept. This process is further reinforced in live-streaming environments, where real-time interaction and shared experiences amplify emotional intensity.

Therefore, integrating the SOR framework with relational and authenticity perspectives suggests a sequential mediation process in which interpersonal interaction influences brand identification through the successive effects of social presence and emotional arousal. This serial indirect effect highlights how socially embedded interaction is translated into relational outcomes through multi-stage psychological processing. Accordingly, the following hypothesis is proposed:

**H8b**. *Interpersonal interaction positively affects consumers’ identification with time-honored brands through the sequential mediating effects of social presence and emotional arousal.*

*Serial indirect effect through brand authenticity and emotional arousal for product interaction.* Within the extended SOR framework, consumer responses are shaped not only by experiential perceptions but also by meaning-based evaluations. In the context of e-commerce live streaming, product interaction serves as a key external stimulus that provides consumers with rich informational and experiential cues. Beyond enhancing immersion, such interaction enables consumers to interpret brand-related meanings, particularly the perceived authenticity of time-honored brands.

Product interaction contributes to the perception of brand authenticity by increasing the transparency and diagnosticity of product-related information. Through real-time demonstrations, detailed explanations, and visual presentations of production processes, consumers are able to directly observe cues related to quality, craftsmanship, and heritage [25,31]. These cues allow consumers to evaluate whether the brand’s current presentation is consistent with its historical identity and core values. As emphasized in brand authenticity theory, such perceived consistency is a fundamental basis for authenticity judgments [1,23].

Brand authenticity, as a meaning-based cognitive evaluation, further triggers affective responses. Prior research suggests that authenticity enhances perceptions of sincerity, credibility, and trustworthiness, which in turn foster positive emotional reactions [38]. In experiential contexts, authenticity is closely associated with emotionally rich responses, as it enables consumers to connect with the symbolic and cultural dimensions of the brand [37]. This process reflects a transition from cognitive evaluation to affective activation within the SOR framework.

For time-honored brands, this mechanism is particularly salient. These brands are inherently associated with historical continuity, cultural heritage, and traditional craftsmanship. When product interaction effectively communicates these elements, consumers are more likely to perceive the brand as authentic and to experience deeper emotional responses, such as nostalgia, cultural resonance, and emotional attachment [25]. Such affective responses represent emotional arousal, which intensifies consumers’ engagement with the brand.

Emotional arousal subsequently facilitates the formation of brand identification by strengthening consumers’ psychological connection with the brand [39,40]. As an affective mechanism, it enables consumers to internalize brand-related meanings and integrate them into their self-concept. This sequential process—from authenticity perception to emotional activation—constitutes a key pathway through which interactive experiences are translated into relational outcomes.

Therefore, integrating the SOR framework with brand authenticity theory suggests a serial indirect effect of product interaction on brand identification through the successive effects of perceived authenticity and emotional arousal. This mechanism highlights the unique role of authenticity as a meaning-based bridge linking interactive stimuli to emotional and relational outcomes in the context of time-honored brands. Accordingly, the following hypothesis is proposed:

**H8c.** *Product interaction positively affects consumers’ identification with time-honored brands through the* sequential mediating effects of perceived brand authenticity and emotional arousal.

*Serial indirect effect through brand authenticity and emotional arousal for interpersonal interaction*. Within the extended SOR framework, interpersonal interaction in e-commerce live-streaming environments represents a socially embedded stimulus that shapes consumers’ internal psychological states through both relational and meaning-based processes. Beyond facilitating communication, interpersonal interaction enables consumers to interpret brand-related meanings, particularly the perceived authenticity of time-honored brands.

Interpersonal interaction contributes to the perception of brand authenticity by enhancing the transparency, credibility, and emotional richness of brand communication. Through real-time dialogue, storytelling, and personalized responses, streamers convey cues related to brand heritage, cultural values, and historical continuity, allowing consumers to evaluate whether the brand’s current representation aligns with its perceived identity [1,23]. Such relational communication reduces psychological distance and increases perceived sincerity, thereby strengthening authenticity judgments [38].

Brand authenticity, as a meaning-based cognitive evaluation, further functions as a precursor to affective responses. When consumers perceive a brand as authentic, they are more likely to experience emotional reactions such as trust, attachment, and psychological comfort [37]. In live-streaming environments, these emotional responses are intensified by the immediacy and interactivity of communication, which enhance consumers’ engagement with brand-related content. This process reflects the transition from cognitive evaluation to emotional activation within the SOR framework.

For time-honored brands, this mechanism is particularly pronounced. These brands are characterized by strong cultural symbolism and historical depth, which can evoke emotionally rich responses when effectively communicated. Interpersonal interaction enables the dynamic presentation of such symbolic elements, allowing consumers to experience the brand’s heritage in a socially embedded context. When authenticity cues are conveyed through interactive storytelling and shared experiences, consumers are more likely to develop emotional resonance, including feelings of nostalgia and cultural identification [23].

Emotional arousal subsequently facilitates the formation of brand identification by strengthening consumers’ psychological connection with the brand [39,40]. As an affective mechanism, it enables consumers to internalize brand meanings and integrate them into their self-concept. In live-streaming contexts, where interaction is immediate and participatory, such emotional processes are further amplified.

Therefore, integrating the SOR framework with brand authenticity theory suggests a sequential mediation process in which interpersonal interaction influences brand identification through the successive effects of perceived authenticity and emotional arousal. This mechanism highlights the unique role of authenticity as a meaning-based bridge linking socially embedded interaction to emotional and relational outcomes in the context of time-honored brands. Accordingly, the following hypothesis is proposed:

**H8d**. *Interpersonal interaction positively affects consumers’ identification with time-honored brands through the sequential mediating effects of perceived brand authenticity and emotional arousal.*

#### 2.2.6. The moderating role of consumer–streamer relationship strength in e-commerce live streaming.

*The moderating role of consumer–streamer relationship strength in the social presence–emotional arousal link.* Within the SOR framework, environmental stimuli influence consumers’ internal psychological states, which subsequently shape behavioral and attitudinal outcomes. While prior hypotheses have focused on the direct and mediating mechanisms linking interaction, social presence, and emotional arousal, these relationships may not be uniform across all consumers. Instead, they are contingent upon relational factors that influence how individuals process and respond to interactive stimuli.

Consumer–streamer relationship strength represents a critical relational boundary condition in e-commerce live-streaming environments. Drawing on social network theory, relationship strength is characterized by the frequency, intensity, and emotional closeness of interactions between individuals [42]. In digital contexts, stronger relational ties are associated with higher levels of trust, familiarity, and emotional attachment, which shape how consumers interpret and respond to communication cues [35].

In live-streaming environments, consumers with strong relationships with streamers—such as loyal followers—are more likely to perceive interactions as authentic, engaging, and personally relevant. This relational closeness enhances their sensitivity to social cues embedded in the environment, thereby strengthening their perception of social presence [30,36]. As a result, when social presence is established, these consumers are more likely to experience intensified emotional responses due to their pre-existing emotional connection with the streamer.

Conversely, consumers with weaker relational ties—such as first-time or casual viewers—may perceive the same level of social presence as less meaningful or engaging. Due to lower familiarity and trust, they are less likely to fully internalize social cues or develop emotional resonance with the content. Consequently, the translation of social presence into emotional arousal is attenuated in such cases.

From the perspective of the extended SOR framework, relationship strength influences the effectiveness of the organismic process by shaping how internal states are activated and transformed into affective responses. Specifically, it determines the extent to which perceived social presence can elicit emotional arousal, thereby functioning as a moderating mechanism in the psychological process.

Therefore, integrating the SOR framework with relational perspectives suggests that consumer–streamer relationship strength moderates the relationship between social presence and emotional arousal. When relationship strength is high, the positive effect of social presence on emotional arousal is strengthened; when relationship strength is low, this effect is weakened. Accordingly, the following hypothesis is proposed:

**H9a**. *Consumer–streamer relationship strength positively moderates the relationship between social presence and emotional arousal.*

*The moderating role of consumer–streamer relationship strength in the brand authenticity–emotional arousal link.* Within the extended SOR framework, brand authenticity represents a meaning-based cognitive evaluation that translates environmental stimuli into affective responses. While prior hypotheses establish the positive effect of perceived authenticity on emotional arousal, this relationship is not uniform across consumers but depends on relational factors that shape how authenticity cues are interpreted and internalized.

Consumer–streamer relationship strength constitutes a critical boundary condition in this process. Drawing on social network theory, relationship strength reflects the frequency, emotional intensity, and trust embedded in interactions between individuals [42]. In digital commerce contexts, stronger relational ties are associated with higher levels of trust, credibility perception, and affective attachment, which influence how consumers process brand-related information [35].

In e-commerce live-streaming environments, consumers with strong relationships with streamers—such as loyal followers—are more likely to perceive streamers as credible and trustworthy sources of information. This trust enhances the perceived reliability of authenticity-related cues, such as brand history, craftsmanship, and cultural narratives, thereby deepening consumers’ evaluation of brand authenticity [1,23]. When authenticity is interpreted within a context of high relational trust, it is more likely to evoke stronger emotional responses, including emotional resonance, attachment, and cultural identification.

Conversely, when the consumer–streamer relationship is weak, consumers may adopt a more skeptical or detached stance toward the information conveyed by streamers. In such cases, authenticity cues are less likely to be fully internalized, as consumers lack sufficient trust and emotional connection to validate the brand’s claims. As a result, the translation of perceived authenticity into emotional arousal is attenuated.

From the perspective of the extended SOR framework, relationship strength influences the effectiveness of the meaning-based mechanism by shaping how authenticity evaluations are transformed into affective responses. Specifically, it determines the extent to which perceived authenticity can elicit emotional arousal, thereby functioning as a moderating factor in the authenticity–emotion linkage.

Therefore, integrating the SOR framework with brand authenticity theory and relational perspectives suggests that consumer–streamer relationship strength moderates the relationship between perceived authenticity and emotional arousal. When relationship strength is high, the positive effect of perceived authenticity on emotional arousal is strengthened; when relationship strength is low, this effect is weakened. Accordingly, the following hypothesis is proposed:

**H9b**. *Consumer–streamer relationship strength positively moderates the relationship between perceived authenticity of time-honored brands and emotional arousal.*

## 3. Research design and data analysis

### 3.1. Data collection and measurement design

#### 3.1.1. Questionnaire design and data collection.

This study used a cross-sectional online survey with purposive non-probability sampling implemented through online convenience recruitment. Ethical approval was obtained from the Institutional Review Board of the School of Business, Anhui University of Technology before data collection commenced (Research Ethics Committee Number: SB-AHUT-REC-2025-06-HS02). Participants were recruited through a questionnaire hosted on the Wenjuanxing platform. Recruitment and data collection were conducted from 2 June to 30 September 2025.

Prior to participation, all respondents were presented with an informed consent statement on the first page of the online questionnaire. The statement explained the purpose of the study, the voluntary nature of participation, the anonymity and confidentiality of responses, and that the data would be used solely for academic research purposes. Participants provided electronic written informed consent by confirming their agreement before proceeding to the questionnaire. Respondents who did not provide consent were not able to continue with the survey. As the target population consisted of consumers aged 18–40 years, all participants were adults, and no parental or guardian consent was required.

Potential participants accessed the questionnaire on mobile devices by scanning a QR code distributed by the researchers. Eligibility screening required respondents to confirm that they were 18–40 years old and had watched e-commerce live-streaming sessions featuring time-honored brands within the preceding six months. Only respondents who met both eligibility criteria were included. Three attention-check items were embedded in the early, middle, and final sections of the questionnaire.

The online recruitment mode was appropriate because the behavior of interest occurs on digital platforms and the study required respondents with recent, context-specific live-streaming experience. Consumers with greater digital access or stronger engagement with live-streaming commerce may have been more likely to participate. Participants were informed that the data would be used solely for academic research. IP addresses were used only to screen for duplicate submissions; names, identification numbers, telephone numbers, and other direct personal identifiers were not collected. After data-quality screening, the analytic dataset contained no information that could directly identify individual participants.

The completed survey data were accessed for research purposes after the survey closed on 30 September 2025. The age criterion reflected the study’s predefined focus on young consumers, and the six-month viewing criterion was intended to reduce recall error by limiting reports to relatively recent live-streaming experiences. These eligibility criteria define the population to which the findings are most directly applicable.

The questionnaire consisted of two main sections. To protect participants’ privacy, demographic information was placed in the second section. The first section included measurement items corresponding to the variables in the research model, while the second section collected demographic information, including gender, age, education level, occupation, frequency of live-stream viewing, and income level. To ensure the reliability and validity of the measurement instrument, the questionnaire items were adapted from established scales in prior studies, with appropriate modifications to fit the research context. Prior to the formal survey, a pilot test involving 30 respondents was conducted. Based on participant feedback regarding ambiguous or unclear items, the questionnaire was revised and refined, resulting in the final version used for data collection. These questionnaire-design procedures—use of established scales, pilot testing to remove ambiguous wording, separation of substantive and demographic items, and ordering of the substantive measures so that the hypothesized causal sequence was not made explicit—also served as ex ante controls intended to reduce common method variance. The corresponding statistical assessment is reported in Section 3.2.1.

Data quality was assessed using duplicate-submission screening, the three attention-check items, and completion time. Responses completed in less than 200 seconds were classified as speeders. Of the 492 questionnaires submitted, 77 did not satisfy the data-quality criteria and were excluded, yielding a final analytic sample of 415 respondents. The demographic characteristics of the final sample are presented in Table 1. The de-identified item-level dataset underlying the analyses is provided in S1 Data.

**Table 1:** Sample demographic characteristics (N = 415).

Variable	Category	Frequency	Percentage (%)
**Gender**	Male	200	48.19
	Female	215	51.81
**Age**	18-25	88	21.20
	26-30	107	25.78
	31-35	137	33.01
	36-40	83	20.00
**Education Level**	Below College Degree	32	7.71
	College Degree	61	14.70
	Bachelor’s Degree	255	61.45
	Master’s Degree	59	14.22
	Doctoral Degree	8	1.93
**Employment Status**	Student	73	17.59
	Employed	279	67.23
	Self-employed	31	7.47
	Unemployed/ Seeking Employment	17	4.10
	Other	15	3.61
**Live-Streaming Viewing Frequency** **(Past 6 Months)**	≥6 times/week	69	16.63
	4–5 times/week	127	30.60
	1–3 times/week	219	52.77
**Monthly Income (**RMB)	≤3000	72	17.35
	3001-6000	76	18.31
	6001-9000	115	27.71
	9001-12000	84	20.24
	≥12001	68	16.39

Sample-size adequacy was evaluated using G*Power 3.1 and the linear multiple regression: fixed model, R² deviation from zero procedure [43]. In line with recommendations to base PLS-SEM sample-size planning on the most complex endogenous regression rather than relying solely on the 10-times rule [44], the most complex equation—predicting emotional arousal from social presence, perceived brand authenticity, consumer–streamer relationship strength, and the two interaction terms—contained five predictors. With an assumed medium effect size (f² = 0.15), α = 0.05, and statistical power (1 − β) = 0.80, the minimum required sample size was 92. The final analytic sample (N = 415) exceeded this benchmark. This calculation supports adequate power to detect a medium overall R² effect in the most complex endogenous equation.

In terms of gender distribution, males accounted for 48.19% and females for 51.81%, indicating a balanced sample. Regarding age, the largest group was 31–35 years (33.01%), followed by 26–30 years (25.78%), while respondents aged 18–25 and 36–40 accounted for 21.20% and 20.00%, respectively. In terms of education, the majority held a bachelor’s degree (61.45%), followed by college degree holders (14.70%) and master’s degree holders (14.22%), while respondents with education below the college level and doctoral degrees accounted for 7.71% and 1.93%, respectively. With respect to employment status, most respondents were employed (67.23%), followed by students (17.59%), with smaller proportions being self-employed (7.47%), unemployed or seeking employment (4.10%), and others (3.61%). In terms of live-stream viewing frequency, the majority reported watching 1–3 times per week (52.77%), followed by 4–5 times per week (30.60%), and 6 or more times per week (16.63%). Regarding monthly income, the largest group earned 6,001–9,000 RMB (27.71%), followed by 9,001–12,000 RMB (20.24%), while those earning 3,000 RMB or less and 12,001 RMB or more accounted for 17.35% and 16.39%, respectively.

#### 3.1.2. Variable definitions and measurement items.

Drawing on prior studies [19,45], this study conceptualizes product interaction and interpersonal interaction as the primary external stimuli within the e-commerce live-streaming context. These interactions are conveyed through multisensory channels (e.g., visual and auditory cues) and serve as the initial triggers of consumers’ internal psychological responses. Accordingly, product interaction and interpersonal interaction are selected as the independent variables.

Because the questionnaire was administered in Chinese and several source instruments were originally developed in English, the relevant items underwent a translation and back-translation procedure before the pilot test. One bilingual researcher translated the original English items into Chinese, and a second bilingual researcher independently back-translated the Chinese version into English. The original and back-translated English versions were compared item by item, and any discrepancies were discussed and resolved to preserve semantic equivalence and the conceptual content of the original measures while adapting the wording to the Chinese e-commerce live-streaming context. The reconciled Chinese version was subsequently evaluated in the 30-participant pilot test described in Section 3.1.1 to assess clarity and contextual appropriateness. Together, these procedures were used to support semantic equivalence and content validity.

Operationally, product interaction was measured as respondents’ perceived access, through the live stream, to product-focused information about usage, technology, and market conditions. Interpersonal interaction was measured as respondents’ active responses to streamer prompts, communication with the streamer, and discussion with other viewers. The measures therefore distinguish an object-centered informational affordance from a person-centered social-exchange affordance. Importantly, the product-interaction items capture information acquisition rather than direct virtual manipulation or explicit craftsmanship verification. Each construct was measured using three items adapted from Pei and Deng (2020) [46].

The mediating variables in this study include social presence, perceived authenticity of time-honored brands, and emotional arousal. Social presence is defined as the extent to which individuals perceive the real presence of others in an online communication environment and was measured using four items adapted from Gong et al. (2023) [47]. Perceived authenticity of time-honored brands refers to the degree to which a brand’s presented attributes align with its core characteristics, historical continuity, and cultural heritage [48]. This construct was measured using six items adapted from the brand authenticity scale developed by Morhart et al. (2015) [49].

The moderating variable, consumer–streamer relationship strength, reflects the frequency, depth, and emotional closeness of interactions between consumers and streamers [42]. This construct was measured using five items adapted from Shi et al. (2005) [50].

Emotional arousal, defined as a deep and instinctive emotional response to external stimuli [51], was measured using four items adapted from Xu and Wang (2023) [5].

The dependent variable is identification with time-honored brands, which captures the positive attitudes and strong emotional bonds that consumers develop toward such brands [52]. This construct was measured using three items adapted from the audience response scale developed by Tuškej et al. (2013) [53].

As shown in Table 2, all measurement items were adapted from well-established scales reported in English- and Chinese-language literature. All constructs except emotional arousal were measured using five-point Likert-type agreement scales ranging from 1 (“strongly disagree”) to 5 (“strongly agree”). Emotional arousal was assessed using four five-point semantic differential items. Higher scores indicated greater levels of arousal.

**Table 2:** Measurement items and sources.

Construct	Code	Measurement Item	Source
Product Interaction	PTIN1	By watching live-streaming content, I can obtain information about product usage (e.g., product features and updates).	Pei and Deng (2020) [46]
	PTIN2	By watching live-streaming content, I can obtain information about product technologies.	
	PTIN3	By watching live-streaming content, I can obtain information about the product’s market situation (e.g., accessories, price, competing products).	
Interpersonal Interaction	ILIN1	While watching live-streaming content, I respond to the streamer’s prompts (e.g., liking, typing “1,” participating in flash sales, commenting, joining lottery draws, grabbing coupons, or purchasing products).	Pei and Deng (2020) [46]
	ILIN2	While watching live-streaming content, I discuss the stream with other viewers.	
	ILIN3	While watching live-streaming content, I communicate with the streamer.	
Social Presence	SLPE1	During this live-streaming session, I feel a sense of human contact.	Gong et al. (2023) [47]
	SLPE2	During this live-streaming session, I experience a sense of sociability.	
	SLPE3	During this live-streaming session, I can exchange information with others.	
	SLPE4	During this live-streaming session, I perceive a sense of human warmth.	
Perceived Authenticity of Time-Honored Brands	ATHB1	Through watching the live stream, I perceive the time-honored brand as a brand with history.	Morhart et al. (2015) [49]
	ATHB2	Through watching the live stream, I perceive the time-honored brand as a timeless brand.	
	ATHB3	Through watching the live stream, I perceive the time-honored brand as an honest brand.	
	ATHB4	Through watching the live stream, I perceive the time-honored brand as a brand that gives back to its consumers.	
	ATHB5	Through watching the live stream, I perceive the time-honored brand as a brand true to a set of moral values.	
	ATHB6	Through watching the live stream, I perceive the time-honored brand as a brand that connects people with what is really important.	
Consumer–Streamer Relationship Strength	SSCR1	I have a strong personal friendship with this streamer.	Shi et al. (2005) [50]
	SSCR2	I have a very close relationship with this streamer.	
	SSCR3	Ending my relationship with this streamer would be costly for me.	
	SSCR4	Switching to another streamer would significantly affect my life.	
	SSCR5	Regardless of circumstances, I feel the need to maintain my relationship with this streamer.	
Emotional Arousal	ELAL1	When watching this live stream, I feel: drowsy — alert.	Xu and Wang (2023) [5]
	ELAL2	When watching this live stream, I feel: calm — excited.	
	ELAL3	When watching this live stream, I feel: inattentive — attentive.	
	ELAL4	When watching this live stream, I feel: unstimulated — stimulated.	
Identification with Time-Honored Brands	ITHB1	I feel that my personality and the personality of this time-honored brand are very similar.	Tuškej et al. (2013) [53]
	ITHB2	I have a lot in common with other people using this time-honored brand.	
	ITHB3	I feel that my values and the values of this time-honored brand are very similar.	

### 3.2. Data analysis

This study employed SmartPLS 4.1.1.8 and SPSS 27 for data analysis, with partial least squares structural equation modeling (PLS-SEM) adopted as the primary analytical technique. Although the proposed model consists exclusively of reflective constructs and could, in principle, be estimated using covariance-based SEM (CB-SEM), the use of PLS-SEM in this study is justified based on several dataset-specific and research-design considerations.

First, the primary objective of this research extends beyond strict theory confirmation to include both explanation and prediction of brand identification with time-honored brands in e-commerce live-streaming contexts. The proposed model incorporates multiple serial mediation mechanisms and moderation effects, forming a moderated chain mediation structure. Prior studies have emphasized that PLS-SEM is particularly appropriate for models focusing on prediction and variance explanation, especially when complex relationships are involved [54]. In this study, brand identification represents a key endogenous construct with substantive theoretical and managerial implications, making predictive capability an important analytical objective.

Second, although all constructs are reflective, the structural model exhibits considerable complexity, including multiple latent variables, indirect effects, and interaction terms. Compared with CB-SEM, PLS-SEM provides greater flexibility and estimation stability in handling complex structural relationships, particularly when mediation and moderation are examined simultaneously [55].

Third, the selection of PLS-SEM is further supported by the distributional characteristics of the dataset. Preliminary analysis indicated that several measurement items deviated from normality in terms of skewness and kurtosis. Since CB-SEM relies on multivariate normality assumptions for maximum likelihood estimation, whereas PLS-SEM is a non-parametric approach, the latter reduces the risk of biased estimates under non-normal conditions [56].

Fourth, the study incorporates latent variable interaction effects to test moderation hypotheses. PLS-SEM allows for the direct estimation of interaction terms using product-indicator and two-stage approaches without imposing restrictive distributional assumptions, making it particularly suitable for modeling moderation within complex structural frameworks [55].

Taken together, although CB-SEM is appropriate for covariance-based theory testing, the present study emphasizes variance explanation, predictive capability, and the estimation of moderated chain mediation effects, making PLS-SEM an analytically appropriate approach aligned with the research objectives and data characteristics.

Following established methodological procedures, a two-stage analytical approach was adopted [57,58]. In the first stage, the measurement model was evaluated to ensure reliability and validity of the constructs. In the second stage, the structural model was assessed to test the hypothesized relationships.

Potential common method bias (CMB) was addressed through design-stage procedural safeguards and post hoc statistical diagnostics; the procedures and results are reported in Section 3.2.1.

To further justify the use of PLS-SEM, the model’s out-of-sample predictive relevance was assessed using the PLSpredict procedure with 10-fold cross-validation [54]. As shown in Table 3, all endogenous constructs exhibit positive Q^2^_predict values, indicating acceptable predictive relevance. Specifically, social presence (Q^2^ = 0.289), perceived authenticity of time-honored brands (Q^2^ = 0.295), emotional arousal (Q^2^ = 0.334), and brand identification (Q^2^ = 0.209) demonstrate positive predictive relevance, suggesting that the model possesses some out-of-sample predictive capability.

**Table 3:** PLSpredict results for endogenous constructs.

Variable	Q²predict	RMSE	MAE
SLPE	0.289	0.848	0.611
ATHB	0.295	0.845	0.617
ELAL	0.334	0.820	0.612
ITHB	0.209	0.894	0.713

Notes: SLPE = Social presence; ATHB = Perceived authenticity of time-honored brands; ELAL = Emotional Arousal; ITHB = Brand identification with time-honored brands.

To further evaluate predictive performance, the root mean square error (RMSE) values of the PLS-SEM model were compared with those of a linear model (LM) benchmark (Table 4). The results indicate that the two models exhibit comparable prediction errors across most indicators, with neither model consistently outperforming the other. According to established guidelines, this pattern suggests moderate predictive power [54].

**Table 4:** Comparison of PLS-SEM and linear model prediction errors (RMSE).

Item	PLS-SEM_RMSE	LM_RMSE
SLPE1	0.870	0.848
SLPE2	0.991	0.976
SLPE3	0.966	0.939
SLPE4	0.924	0.917
ATHB1	0.879	0.879
ATHB2	0.932	0.942
ATHB3	0.865	0.862
ATHB4	0.960	0.950
ATHB5	0.874	0.886
ATHB6	0.861	0.862
ELAL1	0.860	0.863
ELAL2	0.912	0.917
ELAL3	0.963	0.969
ELAL4	0.899	0.879
ITHB1	0.826	0.832
ITHB2	0.934	0.924
ITHB3	0.909	0.885

Notes: SLPE = Social presence; ATHB = Perceived authenticity of time-honored brands; ELAL = Emotional Arousal; ITHB = Brand identification with time-honored brands.

Importantly, the choice of PLS-SEM in this study is not solely driven by predictive considerations but is primarily justified by the complexity of the research model and the exploratory nature of the theoretical framework. The presence of multiple latent constructs, mediation paths, and interaction terms makes PLS-SEM a practical approach for estimating the proposed model. In this regard, PLS-SEM provides significant advantages in handling model complexity and supporting theory development [55].

Overall, although the predictive assessment indicates moderate predictive performance, the use of PLS-SEM remains appropriate and robust for achieving the explanatory and theoretical objectives of this study.

#### 3.2.1. Common method bias assessment.

Because all focal constructs were measured using self-reports collected in a single cross-sectional survey, common method variance could inflate the observed associations. Procedural controls implemented during questionnaire development and administration were therefore combined with post hoc statistical diagnostics [59].

At the procedural stage, respondents were assured that participation was anonymous and confidential and that their data would be used only for academic research; no direct personal identifiers were collected. The substantive measures were adapted from established scales and pilot-tested with 30 respondents, and wording identified as ambiguous or unclear was revised before the formal survey. Demographic questions were placed in a separate section, and the substantive measurement blocks were ordered so that the hypothesized causal sequence was not made explicit. The three attention-check items and completion-time screening were used to identify inattentive responses; these were data-quality screens rather than direct remedies for common method variance.

After data collection, Harman’s single-factor test was used as an initial diagnostic. All 28 substantive measurement items were entered simultaneously into an unrotated principal-components analysis in SPSS 27. The first unrotated component explained 31.562% of the total variance (Table 5), below the conventional 50% benchmark [60–62]. Thus, the test did not indicate that a single general factor accounted for most of the covariance among the items.

**Table 5:** Total variance explained.

Component	Initial Eigenvalues			Extraction Sums of Squared Loadings			Rotation Sums of Squared Loadings		
	Total	% of Variance	Cumulative %	Total	% of Variance	Cumulative %	Total	% of Variance	Cumulative %
1	8.837	31.562	31.562	8.837	31.562	31.562	3.909	13.960	13.960
2	3.452	12.328	43.890	3.452	12.328	43.89	2.958	10.566	24.525
3	1.343	4.797	48.687	1.343	4.797	48.687	2.589	9.246	33.772
4	1.243	4.438	53.125	1.243	4.438	53.125	2.443	8.723	42.495
5	1.123	4.01	57.135	1.123	4.010	57.135	2.129	7.603	50.098
6	1.071	3.824	60.958	1.071	3.824	60.958	2.078	7.422	57.519
7	0.984	3.513	64.472	0.984	3.513	64.472	1.947	6.952	64.472
8	0.876	3.127	67.599						
9	0.739	2.640	70.239						
10	0.701	2.503	72.742						
11	0.608	2.173	74.915						
12	0.590	2.108	77.023						
13	0.548	1.958	78.982						
14	0.532	1.900	80.881						
15	0.522	1.866	82.747						
16	0.486	1.735	84.482						
17	0.474	1.693	86.175						
18	0.471	1.682	87.857						
19	0.434	1.551	89.408						
20	0.424	1.513	90.921						
21	0.388	1.385	92.306						
22	0.365	1.305	93.611						
23	0.351	1.254	94.865						
24	0.332	1.185	96.05						
25	0.309	1.104	97.154						
26	0.286	1.020	98.174						
27	0.269	0.960	99.134						
28	0.243	0.866	100						

As shown in Table 6, the VIF values ranged from 1.000 to 2.024, below conventional thresholds for problematic multicollinearity [63]. Accordingly, the CMB evaluation rests primarily on the procedural safeguards and Harman diagnostic described above. These checks did not reveal a dominant common factor.

**Table 6:** VIF results.

Variable	Tolerance	VIF
ATHB	0.875	1.143
SLPE	0.875	1.143
ELAL	0.494	2.024
ITHB	1.000	1.000

Note: ATHB = Perceived authenticity of time-honored brands; SLPE = Social presence; ELAL = Emotional arousal; ITHB = Brand identification with time-honored brands.

#### 3.2.2. Measurement model assessment.

The measurement model was first evaluated in terms of reliability and validity. As shown in Table 7, all factor loadings ranged from 0.662 to 0.865, exceeding the recommended threshold of 0.60 [64]. Cronbach’s alpha values ranged from 0.729 to 0.896, and composite reliability (CR) values ranged from 0.846 to 0.923, both surpassing the acceptable threshold of 0.70 [65].

**Table 7:** Convergent validity assessment results.

Variable	Items	Factor loading	Cronbach’s α	CR (ρA)	CR (ρC)	AVE
PTIN	PTIN1	0.789	0.732	0.735	0.848	0.651
	PTIN2	0.818				
	PTIN3	0.813				
ILIN	ILIN1	0.842	0.729	0.738	0.847	0.649
	ILIN2	0.759				
	ILIN3	0.814				
SLPE	SLPE1	0.818	0.803	0.806	0.871	0.629
	SLPE2	0.828				
	SLPE3	0.763				
	SLPE4	0.761				
ATHB	ATHB1	0.718	0.817	0.818	0.868	0.523
	ATHB2	0.782				
	ATHB3	0.754				
	ATHB4	0.662				
	ATHB5	0.737				
	ATHB6	0.681				
ELAL	ELAL1	0.788	0.783	0.787	0.86	0.606
	ELAL2	0.815				
	ELAL3	0.724				
	ELAL4	0.783				
SSCR	SSCR1	0.805	0.896	0.902	0.923	0.705
	SSCR2	0.865				
	SSCR3	0.839				
	SSCR4	0.835				
	SSCR5	0.852				
ITHB	ITHB1	0.787	0.729	0.737	0.846	0.647
	ITHB2	0.808				
	ITHB3	0.818				

Notes: PTIN = Product Interaction; ILIN = Interpersonal Interaction; SLPE = Social presence; ATHB = Perceived authenticity of time-honored brands; ELAL = Emotional Arousal; SSCR = Consumer–streamer relationship strength; ITHB = Brand identification with time-honored brands.

Convergent validity was assessed using the average variance extracted (AVE). The AVE values ranged from 0.523 to 0.705. Although the AVE value for perceived authenticity of time-honored brands was 0.523, it still exceeded the minimum acceptable level of 0.50, while the remaining constructs demonstrated higher values [66]. These results indicate that all constructs exhibit satisfactory internal consistency reliability and convergent validity.

Discriminant validity was assessed using three approaches: the Fornell–Larcker criterion, cross-loadings, and the heterotrait–monotrait ratio (HTMT). As reported in Table 8, the square roots of AVE for all constructs were greater than their correlations with other constructs, satisfying the Fornell–Larcker criterion [66]. In addition, the cross-loading results (Table 9) indicate that each measurement item loads more strongly on its corresponding construct than on other constructs, providing further support for discriminant validity [67].

**Table 8:** Discriminant validity assessment using the Fornell–Larcker criterion.

	ELAL	ILIN	PTIN	SLPE	SSCR	ATHB	ITHB
ELAL	**0.778**						
ILIN	0.436	**0.806**					
PTIN	0.356	0.354	**0.807**				
SLPE	0.609	0.499	0.407	**0.793**			
SSCR	0.442	0.265	0.038	0.387	**0.839**		
ATHB	0.554	0.436	0.489	0.603	0.168	**0.724**	
ITHB	0.551	0.404	0.384	0.510	0.329	0.560	**0.804**

Notes: Diagonal elements represent the square roots of the average variance extracted (AVE), while off-diagonal elements represent the correlations among constructs. ELAL = Emotional Arousal; ILIN = Interpersonal Interaction; PTIN = Product Interaction; SLPE = Social presence; SSCR = Consumer–streamer relationship strength; ATHB = Perceived authenticity of time-honored brands; ITHB = Brand identification with time-honored brands.

**Table 9:** Cross-loadings analysis results.

	ELAL	ILIN	PTIN	SLPE	SSCR	ATHB	ITHB
ELAL1	**0.788**	0.324	0.250	0.446	0.293	0.414	0.439
ELAL2	**0.815**	0.339	0.311	0.488	0.377	0.464	0.448
ELAL3	**0.724**	0.363	0.309	0.483	0.181	0.472	0.375
ELAL4	**0.783**	0.334	0.243	0.480	0.498	0.383	0.449
ILIN1	0.395	**0.842**	0.311	0.435	0.167	0.402	0.334
ILIN2	0.287	**0.759**	0.279	0.397	0.251	0.299	0.321
ILIN3	0.364	**0.814**	0.263	0.370	0.232	0.345	0.321
PTIN1	0.277	0.262	**0.789**	0.309	−0.054	0.371	0.285
PTIN2	0.325	0.302	**0.818**	0.357	0.093	0.416	0.333
PTIN3	0.258	0.291	**0.813**	0.316	0.045	0.395	0.309
SLPE1	0.520	0.414	0.324	**0.818**	0.337	0.491	0.441
SLPE2	0.534	0.396	0.289	**0.828**	0.306	0.502	0.428
SLPE3	0.417	0.401	0.328	**0.763**	0.335	0.448	0.447
SLPE4	0.453	0.372	0.355	**0.761**	0.250	0.469	0.299
SSCR1	0.416	0.220	0.060	0.380	**0.805**	0.177	0.319
SSCR2	0.386	0.255	0.069	0.335	**0.865**	0.154	0.317
SSCR3	0.317	0.167	−0.004	0.312	**0.839**	0.077	0.200
SSCR4	0.310	0.201	−0.042	0.233	**0.835**	0.045	0.218
SSCR5	0.400	0.254	0.054	0.340	**0.852**	0.214	0.296
ATHB1	0.350	0.278	0.377	0.393	−0.039	**0.718**	0.368
ATHB2	0.412	0.328	0.368	0.446	0.119	**0.782**	0.416
ATHB3	0.386	0.340	0.387	0.446	0.066	**0.754**	0.405
ATHB4	0.416	0.305	0.295	0.453	0.245	**0.662**	0.400
ATHB5	0.389	0.310	0.362	0.435	0.086	**0.737**	0.401
ATHB6	0.446	0.325	0.333	0.438	0.240	**0.681**	0.435
ITHB1	0.402	0.283	0.254	0.370	0.299	0.396	**0.787**
ITHB2	0.416	0.333	0.289	0.399	0.287	0.453	**0.808**
ITHB3	0.501	0.352	0.371	0.453	0.220	0.494	**0.818**

Notes: ELAL = Emotional Arousal; ILIN = Interpersonal Interaction; PTIN = Product Interaction; SLPE = Social presence; SSCR = Consumer–streamer relationship strength; ATHB = Perceived authenticity of time-honored brands; ITHB = Brand identification with time-honored brands.

To further validate discriminant validity, the HTMT ratios were examined. As shown in Table 10, all HTMT values were below the conservative threshold of 0.85, indicating that the measurement model demonstrates excellent discriminant validity [68].

**Table 10:** Heterotrait–monotrait ratio (HTMT).

	ELAL	ILIN	PTIN	SLPE	SSCR	ATHB	ITHB
ELAL							
ILIN	0.575						
PTIN	0.471	0.482					
SLPE	0.766	0.651	0.531				
SSCR	0.510	0.328	0.099	0.449			
ATHB	0.695	0.560	0.632	0.743	0.221		
ITHB	0.720	0.550	0.516	0.660	0.403	0.719	

Notes: ELAL = Emotional Arousal; ILIN = Interpersonal Interaction; PTIN = Product Interaction; SLPE = Social presence; SSCR = Consumer–streamer relationship strength; ATHB = Perceived authenticity of time-honored brands; ITHB = Brand identification with time-honored brands.

In addition, collinearity among constructs was assessed using variance inflation factors (VIF). As shown in Table 6, all VIF values ranged from 1.000 to 2.024, well below the critical threshold of 5, indicating that multicollinearity is not a concern and that the model exhibits a stable internal structure.

For comparability with earlier PLS-SEM studies, the legacy GoF index was also calculated. The resulting GOF value of 0.47 exceeds the historical large-effect benchmark of 0.36 reported by Wetzels et al. [69].

Overall, the results demonstrate that the measurement model possesses strong reliability, satisfactory convergent validity, and robust discriminant validity, supporting its suitability for subsequent structural model analysis.

#### 3.2.3. Hypothesis testing.

*Direct effects.* The structural model was evaluated using SmartPLS 4.1.1.8. As shown in Table 11, all seven hypothesized direct associations (H1–H7) were statistically significant (all p < 0.001).

**Table 11:** Results of hypothesis testing.

Hypothesis	Path	Standardized Path Coefficient	Standard Error	t-value	p-value	Result
H1	PTIN → SLPE	0.263***	0.057	4.569	0.000	Supported
H2	ILIN → SLPE	0.406***	0.060	6.711	0.000	Supported
H3	PTIN → ATHB	0.383***	0.063	6.021	0.000	Supported
H4	ILIN → ATHB	0.300***	0.060	5.059	0.000	Supported
H5	SLPE → ELAL	0.326***	0.065	4.996	0.000	Supported
H6	ATHB → ELAL	0.317***	0.063	5.009	0.000	Supported
H7	ELAL → ITHB	0.551***	0.042	13.200	0.000	Supported

Notes: PTIN = Product Interaction; SLPE = Social presence; ILIN = Interpersonal Interaction; ATHB = Perceived authenticity of time-honored brands; ELAL = Emotional Arousal; ITHB = Brand identification with time-honored brands. *** p < 0.001.

Product interaction was positively associated with social presence (β = 0.263, p < 0.001), as was interpersonal interaction (β = 0.406, p < 0.001). Product interaction was also positively associated with perceived authenticity of time-honored brands (β = 0.383, p < 0.001), as was interpersonal interaction (β = 0.300, p < 0.001).

Social presence (β = 0.326, p < 0.001) and perceived authenticity of time-honored brands (β = 0.317, p < 0.001) were each positively associated with emotional arousal. Emotional arousal was positively associated with brand identification (β = 0.551, p < 0.001).

*Serial indirect effects.* To test the proposed mediation hypotheses, a bootstrapping procedure with 10,000 resamples was conducted. The results of the mediation analysis are reported in Table 12.

**Table 12:** Results of serial indirect-effect analysis.

Path	Standardized Path Coefficient	Standard Error	t-value	p-value	Result
PTIN → SLPE → ELAL → ITHB	0.047**	0.016	2.968	0.003	Supported
ILIN → SLPE → ELAL → ITHB	0.073***	0.019	3.817	0.000	Supported
PTIN → ATHB → ELAL → ITHB	0.067**	0.020	3.353	0.001	Supported
ILIN → ATHB → ELAL → ITHB	0.053**	0.015	3.426	0.001	Supported

Notes: PTIN = Product Interaction; SLPE = Social presence; ELAL = Emotional Arousal; ITHB = Brand identification with time-honored brands; ILIN = Interpersonal Interaction; ATHB = Perceived authenticity of time-honored brands. ** p < 0.01, *** p < 0.001.

All four hypothesized serial indirect associations were statistically significant. Product interaction showed an indirect association with brand identification through social presence and emotional arousal (β = 0.047, t = 2.968, p = 0.003), supporting H8a, and through perceived brand authenticity and emotional arousal (β = 0.067, t = 3.353, p = 0.001), supporting H8c.

For interpersonal interaction, the corresponding indirect association through social presence and emotional arousal was β = 0.073 (t = 3.817, p < 0.001), supporting H8b, and the indirect association through perceived brand authenticity and emotional arousal was β = 0.053 (t = 3.426, p = 0.001), supporting H8d.

#### These bootstrap estimates are consistent with the proposed serial pathways.

*Moderating effects.* Interaction terms involving consumer–streamer relationship strength were estimated using SmartPLS 4.1.1.8. Neither interaction was statistically significant (Table 13). The SSCR × SLPE term was β = 0.055 (t = 1.013, p = 0.311), and the SSCR × ATHB term was β = −0.049 (t = 0.851, p = 0.395). Thus, the analysis did not detect evidence that relationship strength altered either association in this sample, and H9a and H9b were not supported.

**Table 13:** Results of moderation analysis.

Path	Standardized Path Coefficient	Standard Error	t-value	p-value	Result
SSCR × SLPE → ELAL	0.055	0.055	1.013	0.311	Not Supported
SSCR × ATHB → ELAL	−0.049	0.057	0.851	0.395	Not Supported

Notes: SSCR = Consumer–streamer relationship strength; SLPE = Social presence; ELAL = Emotional Arousal; ATHB = Perceived authenticity of time-honored brands.

**Fig 2:**
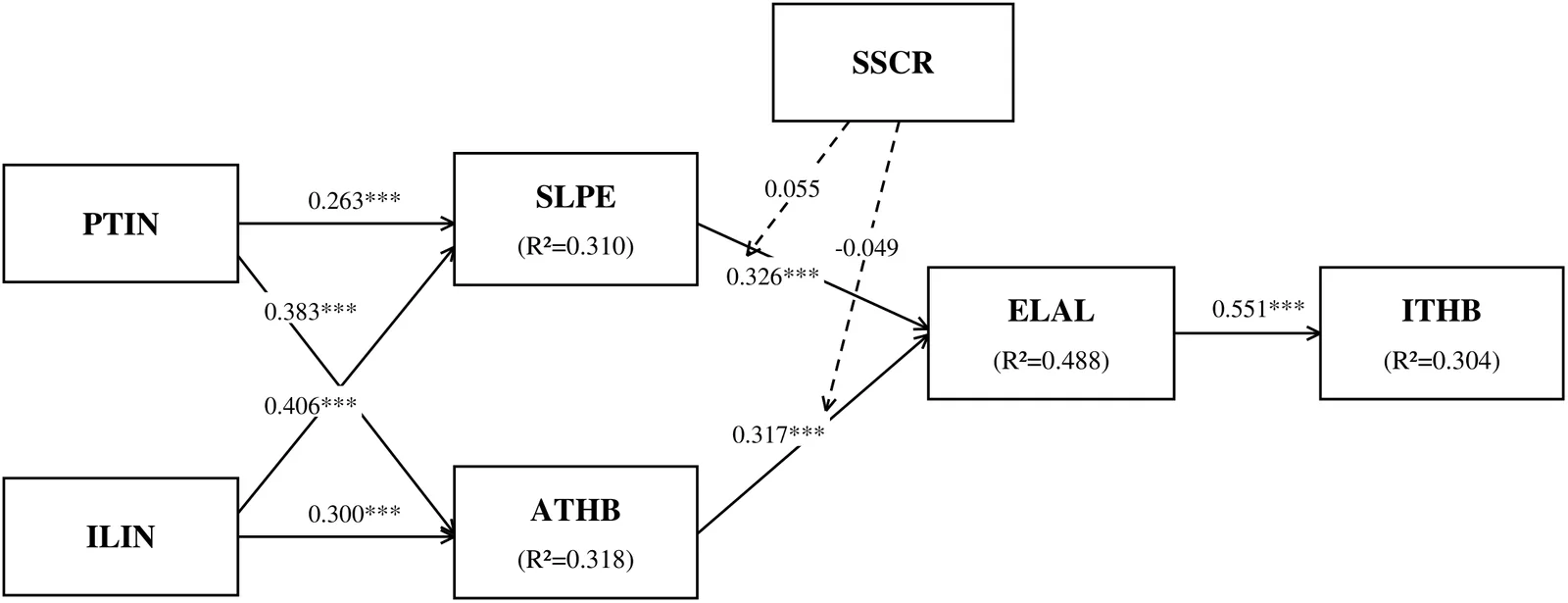
Results of hypothesis testing. PTIN = Product Interaction; ILIN = Interpersonal Interaction; SLPE = Social presence; ATHB = Perceived authenticity of time-honored brands; ELAL = Emotional Arousal; SSCR = Consumer–streamer relationship strength; ITHB = Brand identification with time-honored brands. *** p < 0.001. Solid arrows indicate empirically supported hypotheses, whereas dashed arrows represent unsupported hypotheses.

Explanatory power of the model. The explanatory power of the model was assessed using the coefficient of determination (R^2^). As illustrated in Fig 2, the R^2^ values for social presence, perceived authenticity of time-honored brands, emotional arousal, and brand identification are 0.310, 0.318, 0.488, and 0.304, respectively.

The R² values ranged from 0.304 to 0.488, indicating that the model explained 30.4%–48.8% of the variance in the endogenous constructs. Overall, the results suggest that the proposed model provides a satisfactory level of explanatory capability for the key endogenous constructs in the e-commerce live-streaming context.

## 4. Discussion

### 4.1. Research findings

This study, grounded in the SOR framework and brand authenticity theory, investigates the emotional mechanisms underlying young consumers’ identification with time-honored brands in the context of e-commerce live streaming. Based on 415 valid responses collected from consumers aged 18–40 who had recently engaged with live-streaming sessions of time-honored brands, the empirical analysis was conducted using SmartPLS 4.1.1.8 and the PLS-SEM approach. The study examines how product interaction and interpersonal interaction influence brand identification through social presence, perceived authenticity of time-honored brands, and emotional arousal, as well as the moderating role of consumer–streamer relationship strength.

First, both product interaction and interpersonal interaction were positively associated with social presence, with a numerically larger coefficient for interpersonal interaction (β = 0.406) than for product interaction (β = 0.263). This pattern is consistent with general live-streaming research, but it also clarifies the specific role of social presence in a heritage-brand setting. Wongkitrungrueng and Assarut [25] showed that the utilitarian, hedonic, and symbolic values afforded by live streaming contribute to consumer trust and engagement, while Xie et al. [70,71] identified co-presence and social presence as important components of consumer experience in live marketing. Related research has also shown that streamer type and information-source characteristics shape consumer responses in live broadcasting [72,73]. Zhou et al. [74], from an adoption perspective, further showed that trust is associated with behavioral intention and use of live e-commerce shopping. Whereas these studies primarily connect live-streaming affordances and social experience to trust, adoption, engagement, or purchase-related responses, the present findings place social presence within an affective pathway leading to brand identification. The numerically larger interpersonal-interaction coefficient is consistent with its person-centered character because personalized replies, greetings, and visible exchanges among viewers provide direct cues of reciprocal human presence. Product interaction can also contribute to social presence, although more indirectly, when demonstrations and product-focused answers are delivered contingently by a visible streamer. In time-honored-brand live streams, this social environment may additionally make historically distant craftsmanship and heritage narratives feel contemporaneous and collectively experienced.

Second, both product interaction and interpersonal interaction were positively associated with perceived brand authenticity, with a numerically larger coefficient for product interaction (β = 0.383) than for interpersonal interaction (β = 0.300). This finding is consistent with the core logic of offline authenticity research but extends it to a real-time digital setting. Beverland [75] identified heritage and pedigree, stylistic consistency, quality commitments, relationship to place, production methods, and the downplaying of commercial motives as bases of authenticity in the luxury-wine context. Morhart et al. [49] subsequently conceptualized consumer-based brand authenticity in terms of continuity, credibility, integrity, and symbolism. Case-based research on Chinese time-honored brands has likewise positioned authenticity and value co-creation as central to brand revival [76]. Within this literature, authenticity is generally inferred from accumulated histories, products, practices, and relatively stable forms of communication. Live streaming changes the evidentiary conditions under which these judgments are formed. Viewers can inspect materials and craftsmanship in close-up, request clarification, observe responses to unanticipated questions, and compare brand claims with other viewers’ reactions. Product interaction thus provides relatively direct evidence for evaluating continuity, quality, and craftsmanship, which is consistent with the numerically larger path coefficient. Interpersonal interaction contributes through a different route by providing cues of source credibility, communicative sincerity, and social validation. Live streaming therefore does not replace historical continuity as the basis of authenticity; rather, it makes authenticity claims more immediately observable, contestable, and jointly evaluated under conditions of overt commercialization. However, this interpretation should be bounded by the operationalization used in this study. The product-interaction items assessed access to information about product use, technology, and market conditions rather than direct virtual manipulation or observation of craftsmanship. The empirical result therefore supports an information-diagnosticity route to perceived authenticity; whether close-up craft demonstrations, virtual workshops, or viewer-controlled inspection produce stronger authenticity responses remains to be tested directly.

Third, social presence (β = 0.326) and perceived brand authenticity (β = 0.317) showed similarly sized positive associations with emotional arousal, and emotional arousal was positively associated with brand identification (β = 0.551). This result brings together two streams of prior work that have generally emphasized different outcomes. Live-streaming studies have linked platform affordances to trust and engagement [25], social presence to immersion and other consumer responses [70,71], and trust to adoption and use [74]. Authenticity research, by contrast, has emphasized continuity, credibility, integrity, symbolism, and heritage-based cues as foundations of favorable consumer-brand evaluations [1,23,49,75]. In the present heritage-brand setting, the two mechanisms appear to converge at emotional arousal. Social presence may make the encounter immediate and collectively experienced, while perceived authenticity may provide coherence between the brand’s contemporary presentation and its historical identity. Emotional arousal should therefore not be interpreted solely as excitement produced by the fast pace of live commerce; it may also reflect nostalgia, cultural resonance, and affective investment in the brand’s heritage.

Fourth, the serial indirect-effect results further differentiate these routes. Descriptively, the estimated interpersonal interaction → social presence → emotional arousal → brand identification effect (β = 0.073) was larger than the corresponding product-interaction route (β = 0.047), whereas the estimated product interaction → perceived brand authenticity → emotional arousal → brand identification effect (β = 0.067) was larger than the corresponding interpersonal-interaction route (β = 0.053). This pattern is consistent with the proposed construct boundaries: person-centered exchange is more closely aligned with social presence, whereas object-centered demonstration is more closely aligned with authenticity verification. It also extends prior work that has often examined either general live-streaming value and adoption [25,74] or authenticity as a relatively stable brand evaluation [49,75], rather than modeling how distinct interaction types feed into parallel experiential and meaning-based processes. The significant cross-paths indicate that the mechanisms are complementary rather than mutually exclusive. For time-honored brands, live streaming may reduce distance in two different ways—socially, by making other people feel present, and temporally, by making heritage claims observable and discussable.

Fifth, neither moderating hypothesis was supported. The interaction between consumer–streamer relationship strength and social presence was small and nonsignificant (β = 0.055, p = 0.311), and the interaction between relationship strength and perceived brand authenticity was also nonsignificant (β = −0.049, p = 0.395). These null interaction effects should not be interpreted as evidence that the consumer–streamer relationship is irrelevant. Rather, within the present sample and model, relationship strength did not measurably alter the associations of social presence or perceived brand authenticity with emotional arousal. One possible explanation concerns the cultural and brand-centered nature of the focal context. Time-honored brands derive much of their symbolic value from historical continuity, craftsmanship, and culturally embedded narratives [1,23]. Once consumers perceive a live-streaming encounter as socially vivid or the brand presentation as authentic, these brand-related cues may be sufficiently salient to elicit emotional arousal regardless of the viewer’s prior closeness to the streamer. The streamer may therefore function primarily as a conduit for presenting and interpreting heritage, while the principal object of emotional attachment remains the brand. Under these conditions, consumer–streamer relationship strength may provide little incremental amplification beyond the effects already captured by social presence and perceived brand authenticity. A second explanation concerns the functional location of relationship strength in the proposed process. Social presence and perceived brand authenticity are proximal appraisals of the live-streaming experience, and their links to emotional arousal may operate relatively directly once these appraisals have formed. Relationship strength may instead exert influence at an earlier stage—for example, by affecting whether consumers enter or remain in a live stream, how much attention they allocate to the host, or how readily they form perceptions of social presence and authenticity—rather than by moderating the downstream appraisal-to-arousal links. The unsupported interactions therefore refine the proposed boundary conditions: in heritage-brand live streaming, emotional arousal may depend more on the content and credibility of the brand experience than on pre-existing attachment to a particular streamer.

### 4.2. Theoretical contributions

This study makes several important theoretical contributions to the literature on e-commerce live streaming, consumer behavior, and time-honored brand research.

First, this study extends the literature on time-honored brands by situating brand identification within the emerging context of e-commerce live streaming, with a particular focus on young consumers. Existing studies have emphasized the revitalization of time-honored brands through communication strategies, cross-industry collaboration, and authenticity construction [4,7,10], yet these investigations have largely been confined to traditional marketing environments. With the rapid rise of live-streaming commerce, its unique features—such as real-time interaction, multisensory presentation, and immediate emotional engagement—offer new opportunities for time-honored brands to connect with younger audiences. However, prior research has primarily focused on general consumer behavioral patterns [15,18], while providing limited insights into the emotional mechanisms through which such connections are established. By integrating the live-streaming context with time-honored brand research, this study provides a nuanced understanding of how interactive digital environments can effectively trigger emotional resonance and enhance brand identification among young consumers. In doing so, it enriches the literature on both consumer behavior and the revitalization of time-honored brands in the digital era.

Second, this study develops an integrated theoretical framework that combines the SOR model with brand authenticity theory, thereby offering a more comprehensive explanation of how e-commerce live streaming enhances brand identification. Previous studies have typically relied on single theoretical perspectives—for example, attachment theory to explain purchase intentions [77] or flow theory to examine the effects of interactive features such as real-time comments [78]. While informative, such approaches provide only partial explanations of consumer responses. By contrast, the present study integrates SOR theory with brand authenticity theory to overcome these limitations. Specifically, SOR theory explains how external stimuli (i.e., product and interpersonal interactions) influence consumers’ internal states through social presence, leading to behavioral and emotional responses. However, SOR primarily focuses on the process of response formation and offers limited insight into deep emotional attachment rooted in brand authenticity. The incorporation of brand authenticity theory [1] addresses this gap by emphasizing the role of heritage, cultural embeddedness, and consistency in fostering emotional resonance. This theoretical integration enables a more comprehensive explanation of how consumers develop emotional connections with time-honored brands in live-streaming environments, thereby extending the explanatory power of both SOR theory and brand authenticity theory.

Third, this study advances the literature by identifying dual serial mediation mechanisms involving (1) social presence and emotional arousal, and (2) brand authenticity and emotional arousal. This contribution provides a more fine-grained understanding of how emotional processes shape brand identification in live-streaming contexts. Prior research has predominantly examined factors such as purchase intention [79,80] or streamer characteristics [47], while largely overlooking the role of emotional dynamics in the formation of brand identification. In e-commerce live streaming, consumer decisions are often influenced by non-rational factors, including emotional impulses and social needs. However, these factors have not been systematically incorporated into existing theoretical frameworks. By highlighting the mediating role of emotional arousal, this study fills an important research gap and demonstrates that emotional processes serve as a critical bridge linking interaction stimuli to brand identification. Furthermore, the identification of dual mediation pathways reveals that interaction influences brand identification not only through social immersion but also through authenticity perception, thereby offering a more comprehensive explanation of consumer responses. In addition, although the moderating role of consumer–streamer relationship strength was not supported, this study provides an initial empirical examination of its potential role within the relationships among social presence, brand authenticity, and emotional arousal. This finding suggests that the influence of relationship strength may be more complex than previously assumed, and highlights the need for further theoretical refinement and methodological investigation regarding its boundary conditions in live-streaming contexts.

The unsupported moderation results also qualify the proposed framework. Consumer–streamer relationship strength does not appear to be a universal downstream amplifier of the organismic process. Its role may be stage-specific or context-dependent, becoming more consequential when the streamer’s persona is the focal source of value and less consequential when the streamer primarily communicates a heritage brand’s craftsmanship and cultural meaning. Thus, the null effects narrow the conditions under which relational ties are expected to shape emotional arousal and point to alternative model placements for future research.

### 4.3. Practical implications

The findings suggest that interaction should not be managed as a single, undifferentiated live-streaming tactic. The two pathways imply two operational objectives: product-centered interaction should help viewers verify craftsmanship and heritage claims, whereas interpersonal interaction should make the streamer and other viewers feel responsive and socially present.

First, product interaction should be organized around verifiable evidence of authenticity rather than extended sales narration. Brands can conduct virtual-workshop segments from production sites, use continuous close-up or multi-angle shots to show raw materials, tools, manual techniques, and quality-control checkpoints, and invite master craftspeople to explain why particular processes have been retained or updated. Viewers can request zoom-ins, repetitions, side-by-side comparisons, or product-use demonstrations; relevant traceability, archival, or batch records can also be displayed. When live production is impractical, 360-degree views, virtual try-ons, or brief behind-the-scenes clips can provide comparable evidence. To reduce the commercialization–heritage tension, the evidence segment should precede price promotion, and flash-discount countdowns or scripted urgency should not interrupt craftsmanship verification. Brands should distinguish clearly between retained traditions and modernized production steps, because overstating continuity may undermine the intended authenticity signal.

Second, interpersonal interaction should be designed to create reciprocal recognition and co-presence. Hosts should therefore be trained as heritage storytellers and conversation facilitators rather than operating solely as sales clerks. A session can include scheduled question-and-answer periods, personalized acknowledgment of viewers, prompts inviting consumers to share memories associated with the brand, and direct exchanges between viewers and artisans, archivists, or long-term employees. Hosts can summarize recurring questions, pin substantive answers, respond openly to skeptical comments, and clarify uncertainty rather than rely on uniformly enthusiastic scripts. In high-traffic sessions, a moderator can triage questions so that product, heritage, and service concerns receive timely responses. These practices make the host and co-viewing audience psychologically accessible, thereby targeting social presence more directly than generic exhortations to interact more.

Third, the two interaction types should be sequenced so that information verification and social exchange culminate in emotionally meaningful rather than merely high-intensity experiences. One workable format is to demonstrate the product or craft, interpret its historical and cultural meaning with an artisan or knowledgeable host, invite questions and shared consumer memories, and then present a restrained purchase invitation. Broadcasting from workshops, museums, heritage stores, or other culturally relevant locations, and using archival objects, historical packaging, or production rituals, may evoke nostalgia, pride, curiosity, or admiration. However, emotional storytelling should remain anchored to observable evidence; theatrical excitement disconnected from the product or brand history may reproduce the authenticity dilemma identified in this study. The balance should be adapted to what can be demonstrated safely and credibly in each product category.

Fourth, the unsupported moderation effects indicate that brands should not assume that a strong pre-existing consumer–streamer relationship is required for social presence or authenticity to generate emotional arousal. Managerial resources should therefore be allocated first to brand-centered evidence, host responsiveness, and credible cultural interpretation rather than primarily to celebrity visibility or follower counts. Streams should be intelligible to first-time viewers, for example through a concise introduction to the brand’s history and production standards, while offering deeper craft demonstrations, archive stories, or follow-up communities for returning viewers. Celebrities and influencers may still broaden reach, but their role should be to direct attention to verifiable brand meanings rather than displace the brand as the principal object of identification.

Finally, implementation should be evaluated with pathway-specific indicators rather than sales volume alone. After a live session, brief viewer surveys can assess perceived authenticity, social presence, emotional arousal, and brand identification. Operational indicators can include the proportion of product questions answered, response time, completion and replay rates for craftsmanship demonstrations, participation in question-and-answer segments, saves and shares of heritage content, return-viewer rates, and post-stream community participation. Brands can use controlled A/B tests to compare evidence-first versus discount-first sequencing, artisan-led versus sales-led presentation, or structured versus unstructured question-and-answer formats. Tracking these indicators across repeated sessions would help identify which design choices are associated with stronger authenticity and social-presence responses without treating short-term conversion as the sole criterion of success.

### 4.4. Limitations and future research

Despite these contributions, three limitations qualify the interpretation of the findings. First, the study used a cross-sectional, self-reported survey, although the PLS-SEM model was specified as a sequence of directional relationships. Because all focal variables were measured at one point in time, temporal precedence cannot be established, and reverse causation or omitted-variable explanations remain possible. The estimated paths and serial indirect effects should therefore be interpreted as associations consistent with the proposed framework rather than as causal estimates. Although procedural and statistical controls were applied, residual common method bias also cannot be excluded. Future research should use longitudinal panel designs to examine whether changes in product and interpersonal interaction precede changes in social presence, perceived authenticity, emotional arousal, and brand identification. Laboratory or field experiments could additionally randomize specific interaction features and measure subsequent affective and behavioral responses, thereby providing stronger evidence regarding temporal order and causality.

Second, the geographic and cultural scope of the study is limited to China, where e-commerce live streaming is comparatively mature and time-honored brands carry culturally specific meanings. Platform affordances, norms of streamer-viewer interaction, responses to overt commercialization, and the cultural significance attached to heritage may differ across markets. The findings should therefore not be generalized directly to Western heritage brands or to contexts in which live-streaming commerce is less established without empirical adaptation. In addition, purposive online recruitment may have disproportionately reached consumers who were more digitally connected or more engaged with live-streaming commerce. Cross-national replications should adapt the measures to local heritage-brand meanings, establish measurement invariance, and then compare the structural relationships across cultural and platform contexts.

Third, the analysis pooled time-honored brands across heterogeneous product categories, including food, cosmetics, apparel, and medicine. These categories differ in sensory diagnosticity, perceived risk, regulatory constraints, purchase frequency, and the extent to which craftsmanship can be demonstrated visually. The relative importance of product interaction, interpersonal interaction, and authenticity may therefore vary by category; for example, observable production evidence may be particularly diagnostic in some categories, whereas expert explanation, safety information, or relational reassurance may be more important in others. Because category-specific sample sizes and pathway estimates were not reported, the current coefficients should be interpreted as average associations across product categories. Future studies should recruit sufficiently large stratified samples, test measurement invariance across categories, and use multi-group analysis or interaction models to identify category-specific pathways and managerial implications.

## 5. Conclusions

This study, grounded in the SOR framework and brand authenticity theory, investigates the pathways linking product interaction and interpersonal interaction to young consumers’ identification with time-honored brands in the context of e-commerce live streaming. Based on 415 valid responses collected from consumers aged 18–40 who had recently engaged with live-streaming sessions of time-honored brands, the study employs PLS-SEM to examine the proposed relationships.

The findings showed that product interaction and interpersonal interaction were positively associated with social presence and perceived brand authenticity; both were positively associated with emotional arousal, which was in turn associated with brand identification. Both interaction types also exhibited significant indirect associations through the social presence–emotional arousal and perceived brand authenticity–emotional arousal pathways. The two interaction terms involving consumer–streamer relationship strength were not statistically significant.

From a managerial perspective, the findings support differentiating product-centered and interpersonal strategies. Product-centered segments should prioritize verifiable product information and transparent demonstrations of production and quality, whereas hosts should use responsive question-and-answer exchanges and heritage storytelling to strengthen social presence.

Theoretically, this study contributes to the literature by developing an integrated framework combining SOR theory and brand authenticity theory, and by identifying two statistically significant serial indirect pathways of social presence and emotional arousal, as well as brand authenticity and emotional arousal. These findings provide a novel perspective for understanding how e-commerce live streaming interactions are associated with brand identification, particularly in the context of culturally embedded brands. In addition, the examination of the moderating role of consumer–streamer relationship strength offers preliminary insights into its boundary conditions within emotional mechanisms in live-streaming environments.

The findings should be interpreted in light of the cross-sectional self-report design, purposive online recruitment in China, and pooled product categories. Longitudinal, experimental, cross-cultural, and category-stratified studies are needed to assess temporal ordering, causal mechanisms, and generalizability.

## Supporting information

S1 Data De-identified item-level dataset for the final analytic sample. The file contains the item-level responses and coded demographic variables underlying the analyses reported in this study (N = 415).
(XLSX)
